# Genomic evidence supporting the clonal expansion of extensively drug-resistant tuberculosis bacteria belonging to a rare proto**-**Beijing genotype

**DOI:** 10.1080/22221751.2020.1852891

**Published:** 2020-12-14

**Authors:** Prapaporn Srilohasin, Therdsak Prammananan, Kiatichai Faksri, Jody E. Phelan, Prapat Suriyaphol, Phalin Kamolwat, Saijai Smithtikarn, Areeya Disratthakit, Sanjib Mani Regmi, Manoon Leechawengwongs, Rick Twee-Hee Ong, Yik Ying Teo, Sissades Tongsima, Taane G. Clark, Angkana Chaiprasert

**Affiliations:** a Office for Research and Development, Faculty of Medicine Siriraj Hospital, Mahidol University, Bangkok, Thailand; b Drug Resistant Tuberculosis Research Fund, Siriraj Foundation, Bangkok, Thailand; c National Center for Genetic Engineering and Biotechnology, National Science and Technology Development Agency, Pathum Thani, Thailand; d Department of Microbiology, Faculty of Medicine, Khon Kaen University, Khon Kaen, Thailand; e Research and Diagnostic Center for Emerging Infectious Diseases (RCEID), Khon Kaen University, Khon Kaen, Thailand; f Faculty of Infectious and Tropical Diseases, London School of Hygiene & Tropical Medicine, London, UK; g Division of Bioinformatics and Data Management for Research, Faculty of Medicine Siriraj Hospital, Mahidol University, Bangkok, Thailand; h Research Group and Research Network Division, Faculty of Medicine Siriraj Hospital, Mahidol University, Bangkok, Thailand; i Bureau of Tuberculosis, Department of Disease Control, Ministry of Public Health, Bangkok, Thailand; j Department of Microbiology, Gandaki Medical College Teaching Hospital, Pokhara, Nepal; k Vichaiyut Hospital, Bangkok, Thailand; l Saw Swee Hock School of Public Health, National University of Singapore and National University Health System, Singapore, Singapore; m National Biobank of Thailand, National Science and Technology Development Agency, Pathum Thani, Thailand; n Faculty of Epidemiology and Population Health, London School of Hygiene & Tropical Medicine, London, UK

**Keywords:** Clonal, emerging, MDR, *Mycobacterium tuberculosis*, pre-XDR, proto-Beijing, SNP, whole-genome sequencing, XDR, Clonal expansion of extensively drug-resistant tuberculosis caused by *Mycobacterium tuberculosis* proto-Beijing genotype in Thailand

## Abstract

Tuberculosis disease (TB), caused by *Mycobacterium tuberculosis*, is a major public health issue in Thailand. The high prevalence of modern Beijing (Lineage 2.2.1) strains has been associated with multi- and extensively drug-resistant infections (MDR-, XDR-TB), complicating disease control. The impact of rarer proto-Beijing (L2.1) strains is less clear. In our study of thirty-seven L2.1 clinical isolates spanning thirteen years, we found a high prevalence of XDR-TB cases (32.4%). With ≤ 12 pairwise SNP distances, 43.2% of L2.1 patients belong to MDR-TB or XDR-TB transmission clusters suggesting a high level of clonal expansion across four Thai provinces. All XDR-TB (100%) were likely due to transmission rather than inadequate treatment. We found a 47 mutation signature and a partial deletion of the *fadD14* gene in the circulating XDR-TB cluster, which can be used for surveillance of this rare and resilient *M. tuberculosis* strain-type that is causing increasing health burden. We also detected three novel deletion positions, a deletion of 1285 bp within *desA3* (Rv3230c)*,* large deletions in the *plcB, plcA,* and *ppe38* gene which may play a role in the virulence, pathogenesis or evolution of the L2.1 strain-type.

## Introduction

Tuberculosis (TB), caused by *Mycobacterium tuberculosis*, is one of the top 10 causes of mortality globally. The global problem of drug-resistant TB, especially multi (MDR-TB) and extensive (XDR-TB) forms, is complicating disease control [1]. TB in Thailand remains high with an estimated 153 per 100,000 populations while around 50,167 cases were bacteriologically confirmed in 2018. An estimated 4000 MDR/RR-TB, including 1312 confirmed, were among notified pulmonary TB cases [1]. Treatment for patients with drug-resistant TB is prolonged, expensive and outcomes are poor. Drug resistance in *M. tuberculosis* is almost exclusively due to mutations including single nucleotide polymorphisms (SNPs) in genes coding for drug-targets or -converting enzymes [2,3]. Whole-genome sequencing (WGS) platforms are being used to understand the mutations underlying drug resistance and to characterize transmission, where *M. tuberculosis* isolates (sourced from different hosts) that have near-identical genomic variation are most likely to have arisen from a recent transmission event [4]. Using WGS, molecular epidemiological studies can investigate the cause of emerging drug resistance and focus on the contribution of potential genetic and environmental risk factors across a population to plan for TB control.

The *M. tuberculosis* complex has 8 main lineages (L1-8) [5], which vary in their geographic distribution and spread, with L1 (Indo-Oceanic) and L2 (East Asian) the predominant ones circulating in Thailand [6–10]. The modern Beijing sublineage (L2.2.1; AAF3) has been continuously reported to be associated with clonal expansion of MDR-TB [6,7,11]. In contrast, the “Manu-ancestor” or “proto-Beijing” genotype (spoligotype 523, lineage 2.1; [9]) has a lower prevalence [6,10], with few reports, including detection in the Chiba prefecture in Japan [12] and Guangxi province of Southern China [13]. Recently, we noticed a potential transmission event involving proto-Beijing strains causing XDR-TB [7]. To understand if there is further transmission of the L2.1 strain and any related XDR-TB clonal expansion, we have analysed the WGS from thirty-seven isolates collected over 13-year period from 14 provinces of Thailand.

## Methods

### L2.1 samples and sequencing

Clinical isolates sourced from 725 TB patients across 42 of 77 provinces from Thailand were classified as MDR-TB, pre-XDR, XDR-TB and pan-susceptible by applying phenotypic drug susceptibility testing (DST) assays using drug-containing MiddleBrook 7H10 agar (M7H10). The 723 culturable isolates were from the retrievable stock cultures of 1415 MDR-, pre-XDR and XDR-TB from the Drug-Resistant Tuberculosis Research Fund Laboratory. These isolates were obtained from pulmonary TB patients diagnosed between 2005 and 2012 and covering 42 of 76 provinces in Thailand. All isolates were sub-cultured on Loewenstein-Jensen media and incubated at 37°C for four weeks. Multiple loopfulls of *M. tuberculosis* colonies were taken and DNA extraction was carried out using the cetyltrimethylammonium bromide-sodium chloride method [14]. Genomic DNAs were sequenced using Illumina platforms, across a number of sites, including Novogene AIT (Singapore), Genomic Institute Singapore (GIS), London School of Hygiene & Tropical Medicine, and Mahidol University. We applied the TBProfiler tool [15] to the WGS data to *in-silico* strain-type, and identified 35 (4.8%) isolates as L2.1 strains. To determine the genetic similarity between currently and recently circulating L2.1 causing XDR-TB, two recent L2.1 XDR-TB isolates (2015 and 2017) from Kanchanaburi province were blended into this study. Here L2.1 collection (*n* = 37) represents the largest number of L2.1 strains from Thailand. The raw sequencing data for the 37 samples are deposited in NCBI’s Sequence Read Archive (SRA) database (BioProject ID PRJNA63078). They were analysed together with other 19 L2.1 *M. tuberculosis* strains obtained from public data; 16 isolates from high prevalence area (China), one isolate from the neighbouring country (Laos), and two well-characterized L2.1 isolates from the Sanger Institute [16–19]. The accession numbers of all 56 genomes are listed in Table S1. The 37 L2.1 *M. tuberculosis* were isolated from Thai-patients, across 19 districts among 14 provinces, between 2005 and 2017 by the Drug-Resistant Tuberculosis Research Fund, Faculty of Medicine Siriraj Hospital, Mahidol University, Bangkok (patient no.1–19 and 22–37) and the National Reference Tuberculosis Laboratory (patient no. 20 and 21). Epidemiological linkage between patients was not documented.

### Ethics statement

Approval for the study was given by the Ethical and Scientific Committees of the Faculty of Medicine Siriraj Hospital, Mahidol University (EC No. 510/2561) and the Center for Ethics in Human Research, Khon Kaen University (HE601249).

### Bioinformatics analysis

The L2.1 isolates (*n* = 56) consisted of 37 strains from our collection and 19 globally distributed. The quality of their sequence reads was checked using FastQC (v0.11.7) [20,21]. Sequencing reads were mapped to the *M. tuberculosis* H37Rv reference genome (NC_000962.3) using BWA-MEM software [22]. The sets of mapped paired-end reads were joined using the SAMtools suite. Variants were called using GATK software (v3.8) with HaplotypeCaller in -ERC GVCF mode for generating gVCF files. Multiple gVCF files were aggregated and combined into one GVCF file using GATK GenotypeGVCFs. Only the single nucleotide polymorphisms (SNPs) from VCF output were extracted using SelectVariants tool and filtered using VariantFiltration [23]. The snpEff software (v4.2) was used for variant annotation [24]. SNPs determined in the repetitive regions, paralogous gene families, and drug-resistant genes were discarded using VCFtools [25]. SNPs with allelic frequencies less than 75% or read-depth less than 10 reads were removed. The resulting set of high-confidence SNPs were used to construct a phylogenetic tree based on the maximum likelihood method and 1000 bootstrap replicates using RAxML (v8.0.0) software [26]. iTOL software was used for visualizing the phylogenetic trees [27]. Drug-resistance profiles (and lineages) were predicted *in-silico* using TBProfiler (v2.0) [15]. Spoligotypes were predicted using SpoTyping [28]. The *in-silico* analysis for 31 known regions of differences (RDs) was performed by RD-Analyzer [18]. DELLY software [29] was used to predict large structural variants (SVs) using minimum paired-end mapping quality 20 and standard deviation units as 3 times. High-quality SVs had to be supported by paired-end and split reads. All variants across the samples were merged into a single file using BCFtools software. All putative genetic markers were manually reviewed for accuracy using Integrated Genomic Viewer software [30], and, if considered as true variant they were included for delineation.

## Results

### Setting and expansion of *M. tuberculosis* L2.1 in Thailand

**Figure 1.**
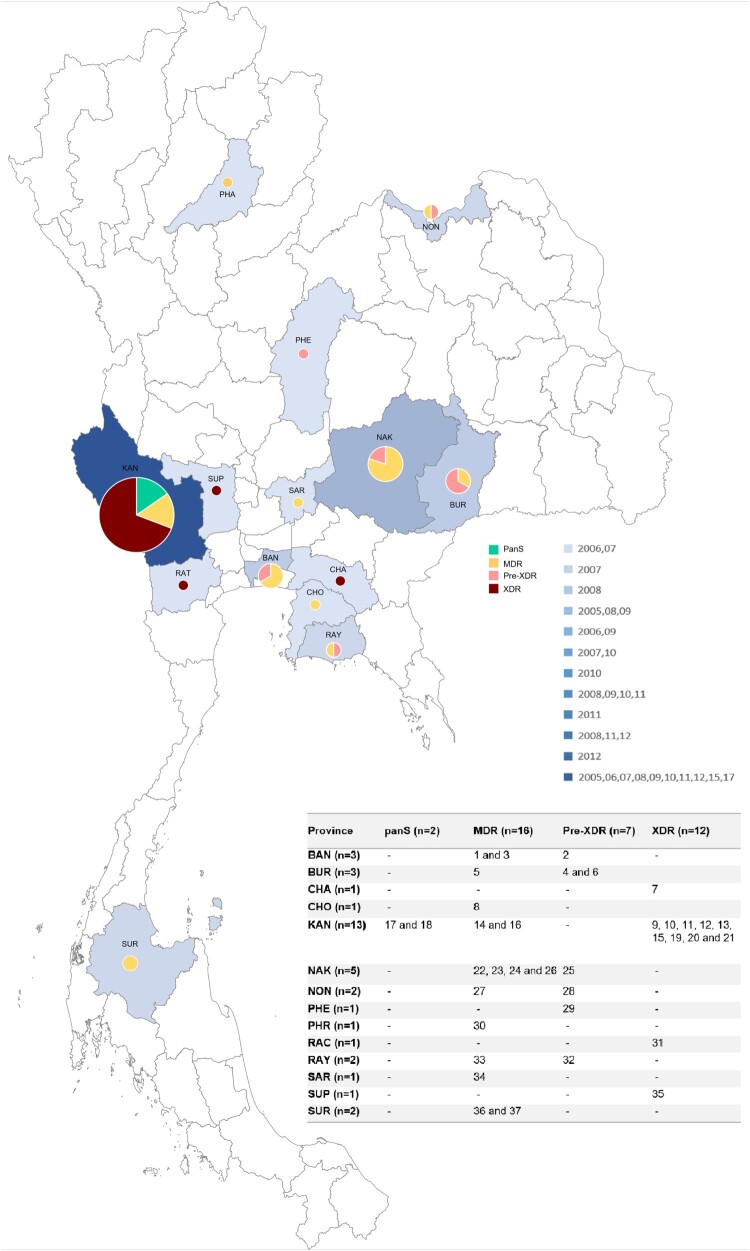
Geographic distribution of pan-susceptible (green), MDR-TB (yellow), pre-XDR (pink), and XDR-TB (dark red) caused by *M. tuberculosis* L2.1. Each province has been shaded according to the frequency of sample collection including Bangkok (BAN), Buriram (BUR), Chachoengsao (CHA), Chonburi (CHO), Kanchanaburi (KAN), Nakhonratchasima (NAK), Nongkhai (NON), Phetchabun (PHE), Phrae (PHR), Ratchaburi (RAT), Rayong (RAY), Saraburi (SAR), Suphanburi (SUP), and Suratthani (SUR).

The 37 clinical *M. tuberculosis* strains used were isolated from pulmonary TB patients in 14 provinces from 2005 to 2017. In 2005, the L2.1 sub-lineage was found only in Kanchanaburi and Bangkok. Seven years later, L2.1 were found across 14 provinces and 5 were XDR-TB (Figure 1).

The overall rate of MDR-TB individuals with pre-XDR and XDR-TB are high. Twelve isolates (32.4%) are XDR-TB whereas 7 isolates (18.9%) are pre-XDR. Sixteen isolates are MDR-TB (43.2%) and the remaining 2 isolates are pan-susceptible. Demographic data of each patient is shown in (Table 1 and Table S1). The L2.1 incident rate (*n* = 37 or 5.1%) was high compared with studies in the Chiangrai province, Northern Thailand (1.0%) [10] and Guangxi province of Southern China (0.2%) [13]. Unfortunately, our collection had no isolates from Chiangrai. Within province the highest number of L2.1 strain was observed in Kanchanaburi (*n* = 13), followed by Nakhon Ratchasima (*n* = 5), Buriram and Bangkok (*n* = 3) province.

**Table 1.** SVs identified among the L2.1 sub-population. The position is relative to the *M. tuberculosis* H37Rv reference genome (NC_000962.3).

Uniqueness	SV types	Start	Stop	Genes	Gene symbols and descriptions
MDR-TB cluster	Deletion	2900282	2900432	Rv2576c	membrane protein
	Deletion	2900903	2901086	Rv2577	hypothetical protein_metallophosphatase superfamily
Pre-XDR cluster	Deletion	1013430	1015391	Rv0909 Rv0910	Antitoxin toxin
	Deletion	2851350	2851718	Rv2527	*vapC*17: ribonuclease
	Deletion	3071595	3078945	Rv2761c Rv2762c Rv2763c Rv2764c Rv2765 Rv2766c Rv2767c Rv2768c Rv2769c	*hsdS*: type I restriction/modification system specificity determinant hypothetical protein *dfrA*: dihydrofolate reductase *thyA*: thymidylate synthase hydrolase f*abG5*: short-chain type membrane protein *PPE43*: PPE family protein PPE43 *pe27*: PE family protein PE27
XDR-TB cluster	Deletion	1180695	1180773	Rv1058	*fadD14*: fatty-acid–CoA ligase
All isolates	Deletion	2010609	2011436	Rv1777	*cyp144*: cytochrome P450
	Deletion	3606557	3607841	Rv3229c Rv3230c	*desA3*: stearoyl-CoA 9-desaturase stearoyl-CoA 9-desaturase electron transfer protein
	Deletion	2629526	2634350	Rv2350c Rv2351c Rv2352c	*plcB*: membrane-associated phospholipase B *plcA*: membrane-associated phospholipase A *PPE38*: PPE family protein PPE38

### Genotyping analysis of the rare genotype L2.1 isolates in Thailand

**Figure 2.**
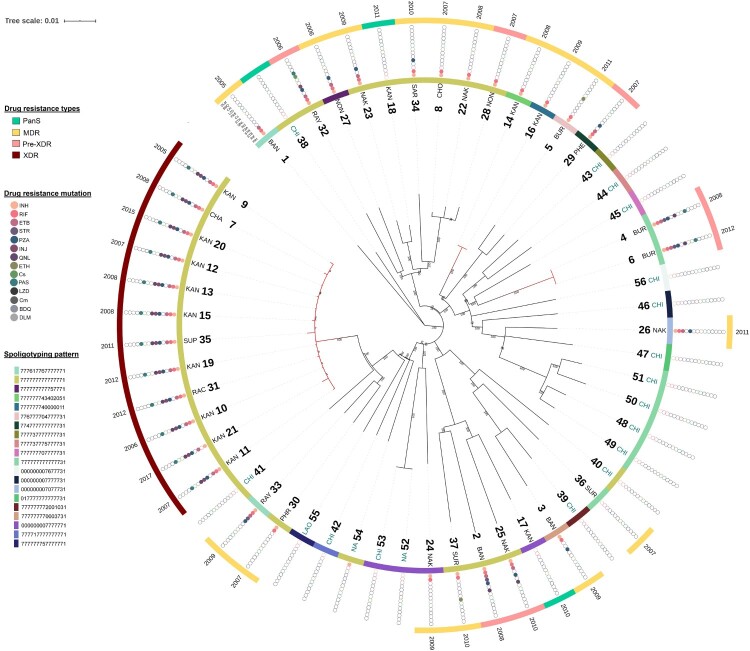
Genetic diversity and distribution of 56 *M. tuberculosis* L2.1 strains. Phylogenetic tree based on maximum likelihood method using 3685 high-confidence SNPs with their collection site, spoligotyping pattern drug-resistance phenotype, drug-resistance mutation, and year of collection (from inner to the outer circles). The red branch indicated the potential transmission clusters.

The genotypic drug-resistance profile of each isolate is shown in Figure 2. *In silico* spoligotyping classified the 37 L2.1 strains into 12 genotypes, and the most common spoligotype (23/37; 62.2%) was SIT 523 (777777777777771). Of these SIT 523 isolates, 12 (52.2%) were XDR-TB, and were sourced from 4 provinces; Chachoengsao (*n* = 1), Kanchanaburi (*n* = 9), Ratchaburi (*n* = 1), Suphanburi (*n* = 1) (Figures 1 and 2). All isolates harboured the extended RD105 deletion but had RD207, RD181 and *pks15/1* regions intact.

To investigate the within and between country spread of L2.1, an analysis of the genome variation of all 56 isolates (Thailand, China, Laos, and others) was performed. A total of 3685 high-confidence SNPs were identified across 56 *M. tuberculosis* isolates, including 3206 (87.0%) in coding regions (1141 silent, 2028 missense, and 37 nonsense), 12 in non-coding and 467 in intergenic regions (Table S2). Across the 56 isolates, 2484 (67.4%) SNPs were identified in single isolates. A phylogenetic tree based on the 3685 SNPs identified revealed that Thai-isolates were mostly interleaved with other countries including China (CHA), Laos (LAO), and others (NA) (Figure 2). All 12 (100%) XDR-TB isolates were clustered together and were distinguished from the others by at least 47 SNPs. Twenty-seven of 47 SNPs had missense mutations. This large and long-lasting monophyletic clade with high bootstrap support (100%) included XDR-TB cases from Kanchanaburi (*n* = 9). The clade was also interleaved with XDR-TB isolates from Ratchaburi (*n* = 1), Chachoengsao (*n* = 1), and Supanburi (*n* = 1) (Figure 3(A)). The time range between the first and last isolate was 13 years (2005–2017). Moreover, the results revealed that 2 (28.6%) pre-XDR and 2 (12.5%) MDR-TB patients identified were limited to one geographic district and separated by at least 90 SNPs and 60 SNPs, respectively, from any other study isolates. This result implies that this particular strain was circulating locally. A genotypic clustering rate of 43.2% was found among patients who were infected with L2.1 strain, revealing the high level of transmission in this population. Figure 3.(A) Map of Thailand annotated with the collection site of MDR- (dark green), pre-XDR (pink), and XDR-TB (dark red) cluster; (B) Frequency of pairwise genetic distance among 37 samples from Thai-patients; pan-susceptible (light blue), MDR-TB (dark green), pre-XDR (pink), and XDR-TB (dark red).

**Figure:**
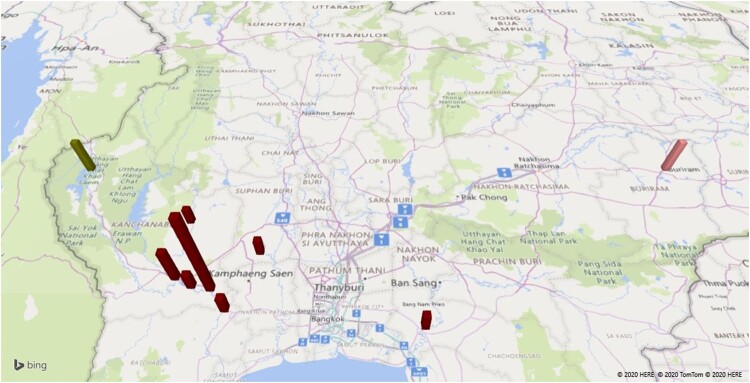

**Figure:**
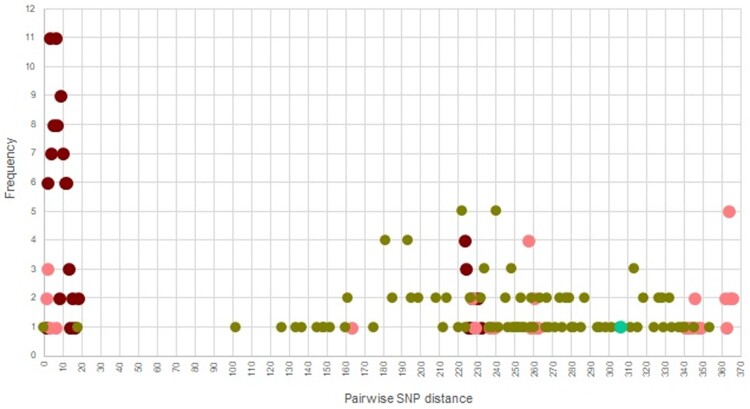

### Evidence of transmission

Potential transmission clusters were revealed by determining the pairwise SNP distance between the 37 Thai-isolates. The pairwise genetic distance varied between 0 and 381 SNPs (Table S3). Using a 12 SNP cut-off [31], 43.2% of patients belonged to 3 different possible clusters, including: (i) an XDR-TB cluster with a maximum size of 12 isolates (Figure 3(B)), (ii) 2 pre-XDR isolates, and (iii) 2 MDR-TB isolates. Mutation patterns associated with resistance were also unique for each cluster (Table S4), providing potential evidence of transmission. The MDR-TB cluster (*n* = 2; no.14 (2008) and 16 (2009)) was composed of patients from the same district and had identical SNP patterns, including mutations known to be responsible for drug resistance (*rpoB* Ser450Leu; *katG* Ser315Thr). Within the pre-XDR cluster (*n* = 2; no.4 (2008) and 6 (2012)), there was only 1 SNP difference (a synonymous mutation difference between isolates 4 and 6), and the established drug-resistance mutations were identical (*katG* Ser315Thr; *rpoB* Ser450Leu; *rpsL* Lys88Arg; *embB* Gly406Asp; *pncA* Tyr103His; *gyrA*: Asp94Asn; *thyA* deletion 3071595-3077080). For the XDR-TB cluster (*n* = 12), the pairwise genetic distance varied between 1 and 11 SNPs. Nine (75.0%) XDR-TB were isolated from Kanchanaburi province, including Thamaka (*n* = 5; no.9 (2005), 12 (2007), 15 (2008), 20 (2015), and 21 (2017)), Muang (*n* = 2; no.11 (2007) and 13 (2008)), Bophloi (*n* = 1; no.10 (2006)) and Thamuang (*n* = 1; no.19 (2012)) districts. The other three isolates collected from patient in Chachoengsao (no.7 in 2008), Rachaburi (no.31 in 2012), and Suphaburi (no.35 in 2011). Ten (83.3%) XDR isolates had identical resistance mutation profiles (*katG* Ser315Thr; *rpoB* Ser450Leu; *embB* Met306Ile; *pncA* Ile90Ser; *rrs*: 1401a > g; *gyrA* Asp94Gly; *folC* Glu40Gly). XDR-TB samples from Chachoengsao (no.7), and Suphanburi (no.35) were located 174 and 64.7 kilometres, respectively, away from earlier isolates. They had identical drug-resistant mutations to the majority of the XDR-TB cluster, except for the addition of the Leu527Val mutation in the (rifampicin compensatory) *rpoC* gene. The recent isolate (no. 20; year 2015) had an additional mutation (*thyX* −16C > T) related to para-aminosalicylic acid resistance. These results suggest that there was a long-term transmission of local clusters of XDR-TB, which carried common drug-resistance mutations, thereby explaining its success as a spreader [32–34].

During the study period, the first XDR-TB case (patient no. 9) was infected with an L2.1 XDR-TB clone from Kanchanaburi in 2005. Subsequent events have revealed that the XDR-TB clone has emerged continuously, of which 12 had at least one isolate over a span of 13 years, and been restricted to within 180 km across four provinces from the first case.

### Structural diversity in clonal cluster population

Structure variations (SVs) were called relative to the *M. tuberculosis* H37Rv reference genome. Thirty-nine high-quality SVs were detected. Three common deletion regions across all combined dataset of L2.1 isolates were detected with sizes between 827 and 4824 bp (Table 1), all exclusive to the sub-lineage. The largest genome SV involved a deletion of *plcA* (Rv2351c) and *PPE38* (Rv2352c), and a partial deletion of *plcB* (Rv2350c). There were deletions unique to the clusters (MDR-TB 2; pre-XDR 2). Twelve isolates belonging to the XDR-TB cluster contained one unique deletion in the Rv1058 region, which was not found in any other isolates.

## Discussion

The recent emergence of drug-resistant TB infections in Thailand has been attributed to strains in *M. tuberculosis* lineage 2, particularly the “modern Beijing” (Lineage 2.2.1) sub-lineage. *M. tuberculosis* from this sub-lineage are highly virulent causing disease outbreaks, with escape from the effects of BCG vaccination, they disseminate efficiently, and easily acquire antibiotic resistance [6,7,9,35]. The more rare “proto-Beijing” (L2.1) appears to be transmitting, and has a high propensity to be XDR-TB. In our study, the number of MDR-TB individuals with XDR-TB is ∼6 times greater than the WHO global estimate (6.2%) [1], and all are clustered. Poor treatment adherence is not the only factor that contributes to drug resistance, but also the failure in controlling transmission of drug-resistant strains.

This study was a hospital-based retrospective analysis included the retrospective and passive nature of case-finding, based only on retrievable isolates from stock cultures during 2005–2012; therefore, they were a set of convenience isolates and may not be representative of all isolates from the community, and we cannot exclude some unintended bias. Additionally, TB database information of Thailand during 2005–2012 was not well developed, and the capacity of the laboratory to perform phenotypic drug susceptibility testing was also limited. However, the number of L2.1 isolates retrieved is the largest to date from a single country, and the underlying sampling from 725 culturable *M. tuberculosis* covers 42 of 77 provinces [2,6,7,17]. Here we reported the presence of L2.1 isolates, including in Bangkok, Buriram, Chachoengsao, Chonburi, Kanchanaburi, Nakhonratchasima, Nongkhai, Phetchabun, Phrae, Ratchaburi, Rayong, Saraburi, Suphanburi, and Suratthani, but not Chiangrai provinces [7,10].

WGS data were integrated with routinely clinical information to identify the putative TB cluster. Unfortunately, epidemiological information was not available in this study. The integration of social-network analysis with high-resolution bacterial genome sequencing would provide traceable information in evaluating TB transmission [36]. The 12-SNP distance was proposed by using studies in low incidence TB settings [31], and seems reasonable for our study, set in the contexts of low L2.1 population prevalence and the community transmission investigation. Using clustering threshold of 12 SNPs, we defined 3 distinct unique clonal clusters which carried particular drug-resistant mutation pattern for each clone (Table S4), implying diverse evolution histories for the individual population. A high clustering rate of 43.2% among L2.1 infected patients highlighted the high transmissibility of proto-Beijing strains. Two of the three clusters identified had one pair of strains and emerged in a particular region. Among the largest possible cluster groupings consisting of twelve XDR-TB transmission clusters, at least seven putative transmission events were postulated to underlie the largest cluster on the basis of a shared social setting and WGS genotyping, in which each individual isolate was supposed to be accumulated genetic diversity within-host [31]. Nine isolates were retrieved from patients in Kanchanaburi province, of which four district hospitals were involved in four putative transmission events; 5 (41.7%) isolates from Thamaka, 2 isolates from Muang, 1 isolate from Tha muang, and 1 isolate from Bo phloi district. The other 3 events were possibly infected through social contact in Chachoengsao (patient no.7), Rachaburi (patient no.31), and Supanburi (patient no.35). However, the potential of clonal expansion reported here would switch attention towards a rare genotype causing XDR-TB in Kanchanaburi province, not only in a particular hospital but through the high prevalence of MDR-TB, especially within L2.2.1; AAF3 [7,11,36,37]. The lack of transfer of these hyper-resistant strains to other geographical locations may be associated with adaptation to local-host population, as seen in L1 strains [9] or other factors which need to be explored in the future.

Forty-seven SNPs and a 78 bp deletion in *fadD14* (encoded fatty-acid–CoA ligase) were present in all XDR-TB clonal spreaders, but not other isolates studied. Notably, 27 (of the 47) SNPs resulted in a missense mutation and were potentially subject to natural selection. The genetic markers shared by the successful XDR-TB cluster could be related to virulence or used to screen for the existence of antibiotic-resistant bacteria. Specific resistance-conferring mutations and strain genetic background are often associated with a fitness cost [32,33]. The local clusters of XDR-TB carried common drug-resistance mutations, potentially explaining its successful spreading phenotype more readily than other genotypes. Unavoidably, host genetic background also influences the response to mycobacterial activity [6,17]. The successive appearance of XDR-TB cluster conferred overgrowth their ancestral drug-susceptible clone that become undetectable in this study. Mutations found within the XDR-TB cluster indicate a remarkable adaptation capacity during infection. Further, the factors influencing transmission remain poorly understood, but as WGS becomes routine in a clinical setting, it will be possible to reconstruct the transmission chains and assess the host and *M. tuberculosis* genetic and non-genetic factors affecting transmissibility.

We detected three novel deletion positions which may play a role in the virulence, pathogenesis or evolution of the L2.1 strain-type. Poorer bacterial growth has been observed in presence of azole in *M. tuberculosis* strains with *CYP144* knockout [38]. Azole drugs may have activity against the L2.1 strain. Further, a deletion of 1285 bp within *desA3* and oxidoreductase Rv3230c may cause the L2.1 strain to be resistant to the second-line anti-TB drug isoxyl [39]. Large deletions in the *plcB, plcA,* and *ppe38* gene were also detected. The deletion of *ppe38* and loss of ESX-5 substrates secretion as found in modern Beijing sub-lineages may have increased their virulence and contributed to their global spread [40]. Whereas partial deletion of phospholipase C encoded by *plc* genes (*plcA, B, C* and *D*) may have a role in assisting the bacteria to escape from phagosomal containment, as found in *Listeria monocytogenes* or *Clostridium perfringens* [41]. This may cause lower virulence than a modern Beijing sub-lineage.

Overall, our study has revealed that *M. tuberculosis* sub-lineage L2.1 is geographically restricted but has a great propensity to be XDR-TB and transmit. We anticipate that revealing the XDR-TB burden of sub-lineage L2.1 will lead to more widespread surveillance and much-needed epidemiological studies. The strong recommendation from the Thailand national TB control program to implement N95 or equivalent mask wearing for active TB patients especially MDR-, pre-XDR and XDR-TB patients will be an additional measure to prevent TB transmission during intensive treatment.

## Supplementary Material

Supplement_2020208_PrS.xlsx
